# Clinical features and outcomes of Myasthenia Gravis associated with COVID-19 vaccines: A systematic review and pooled analysis

**DOI:** 10.1097/MD.0000000000034890

**Published:** 2023-10-06

**Authors:** Amir Hossein Tayebi, Parham Samimisedeh, Elmira Jafari Afshar, Aryan Ayati, Elaheh Ghalehnovi, Laleh Foroutani, Nahid Abbasi Khoshsirat, Hadith Rastad

**Affiliations:** a Cardiovascular Research Center, Alborz University of Medical Sciences, Karaj, Iran; b Faculty of Medicine, Tehran University of Medical Sciences (TUMS), Tehran, Iran; c Razi Vaccine and Serum Research Institute, Karaj, Iran; d Department of Neurology, Shahid Rajaei Hospital, Alborz University of Medical Sciences, Karaj, Iran.

**Keywords:** autoimmune disorder, clinical features, COVID-19 vaccine, Myasthenia Gravis, prognosis

## Abstract

**Backgrounds:**

Myasthenia Gravis (MG), a chronic neuromuscular junction disorder, emerged as one of the serious side effects of the Coronavirus Disease 2019 (COVID-19) vaccination. We aimed to summarize the findings of studies on the clinical features and outcomes of COVID-19 vaccination-associated MG.

**Methods:**

We performed a systematic search on 3 databases, Medline, Embase, and Scopus, using the query “COVID-19 vaccine” and “Myasthenia Gravis.” Patients’ data, including clinical data, MG subtype, vaccine type, and vaccine dose number, were extracted from the eligible studies.

**Results:**

A total of 20 COVID-19 vaccination-related MGs have been reported worldwide. The median (interquartile range) age was 64 (51, 75) years; 85% (17/20) of them were male, and 70% (14/20) of patients had received messenger RNA-based vaccines. The most common symptoms, in order of frequency, were binocular diplopia (8/11) and ptosis (4/11); the median (interquartile range) time from vaccine to MG symptoms was 6 (2, 7.5) days. Repetitive nerve stimulation showed abnormal decrement in 85% (11/13) of patients, and all 4 patients getting single-fiber electromyography showed an abnormal finding. Nine out of twelve patients with data on clinical outcomes experienced partial/complete improvement of symptoms within 1 month.

**Conclusion:**

MG cases after the COVID-19 vaccine are more likely to occur among males and adults older than 50 years. Our pooled cohort data suggest MG symptoms appear within 2 weeks after receiving the vaccine. The presenting symptoms in MG cases associated with COVID-19 vaccine are possibly similar to non-vaccination related MGs. Most patients are expected to experience partial/complete improvement within 1 month.

## 1. Introduction

Vaccination remains the essential strategy for controlling the Coronavirus Disease 2019 (COVID-19) pandemic in the long term.^[1]^ To this date, more than 12.8 billion shots of vaccines have been administered globally^[2]^; however, as the immunity from these vaccines diminishes over time, vaccine booster doses are needed to maintain protection.

Questions and hesitancy concerning the safety and vaccine-induced autoimmune diseases^[3–5]^ have been raised. A growing number of cases developed inflammatory side effects, including neuromuscular junction disorders, shortly after COVID-19 vaccination worldwide.^[6]^ Myasthenia Gravis (MG), a chronic neuromuscular junction disorder, emerged as a serious side effect of the COVID-19 vaccination. While MG is rare, a late diagnosis can result in a myasthenic crisis and even death.

Increasing case reports/series have recently described the symptoms and clinical course in patients with COVID-19 vaccination-associated MG. However, they are limited by their small sample size. We summarized and analyzed available evidence to enhance the current understanding of the characteristics, course, and prognosis of the COVID-19 vaccine-associated MG.

## 2. Method

We conducted this systematic review according to the Preferred Reporting Items for Systematic Reviews and Meta-Analyses statement. As a review of published articles, no ethical approval or patient consent was required. We included all eligible studies that assessed clinical features and outcomes in patients with COVID-19 vaccine-associated MG.

### 2.1. Search

We performed a comprehensive search on 3 databases, Medline, Embase, and Scopus, on November 12, 2022, and updated it on January 6, 2023, using a combination of key terms in 2 domains, including “COVID-19 vaccine” and “Myasthenia Gravis.” Also, we manually checked the reference lists of the relevant articles for any additional eligible studies. We imported all retrieved citations into EndNote X9 and removed the duplicate items.

### 2.2. Inclusion process and criteria

Two researchers [AT and PS] independently screened the titles, abstracts, and full texts of the retrieved studies to include the eligible items. We included all studies that met all our eligibility criteria:

1. Reporting incident case(s) of MG following COVID-19 vaccination.
2. Having an observational design: cohort studies, case series, and case reports.
3. Written in English.

### 2.3. Data extraction

After retrieving the eligible studies, 2 researchers [AT and EJ] extracted the data from the retrieved articles into a predefined data extraction form in Microsoft Excel (version 2016, Microsoft Corp., Redmond, WA). The extracted data included: the first author’s name, year, country, age, and gender, clinical data, MG subtype, vaccine type, vaccine dose number (first, second, booster), the time between injection and symptom onset, presenting signs and symptoms, electrophysiological findings, laboratory data, treatment, and patient follow up.

### 2.4. Quality assessment

The quality of the included case series was assessed independently by 2 trained researchers (PS and EGH) using the Joanna Briggs Institute appraisal tool adapted for case series^[7]^; any disagreement was resolved through discussion with a third researcher (HR or AT). The Joanna Briggs Institute appraisal tool included 10 items, each receiving 1 score (Table S1, Supplemental Digital Content, http://links.lww.com/MD/J598).

### 2.5. Statistical analyses

We used Microsoft Excel (version 2016, Microsoft Corp., Redmond, WA) to organize the extracted individual patient data and to produce tables. We integrated the individual patient data case series and case report into a single case series. A valid percent was calculated for each categorical variable by excluding cases without data for the characteristic of interest from the denominator. Also, continuous variables were summarized using the median (interquartile range) or mean and standard deviation (SD). Data analysis was conducted using Stata/MP Version 16 (Stata Corp. LP, College Station, TX/METAN package).

## 3. Results

**Figure 1.**
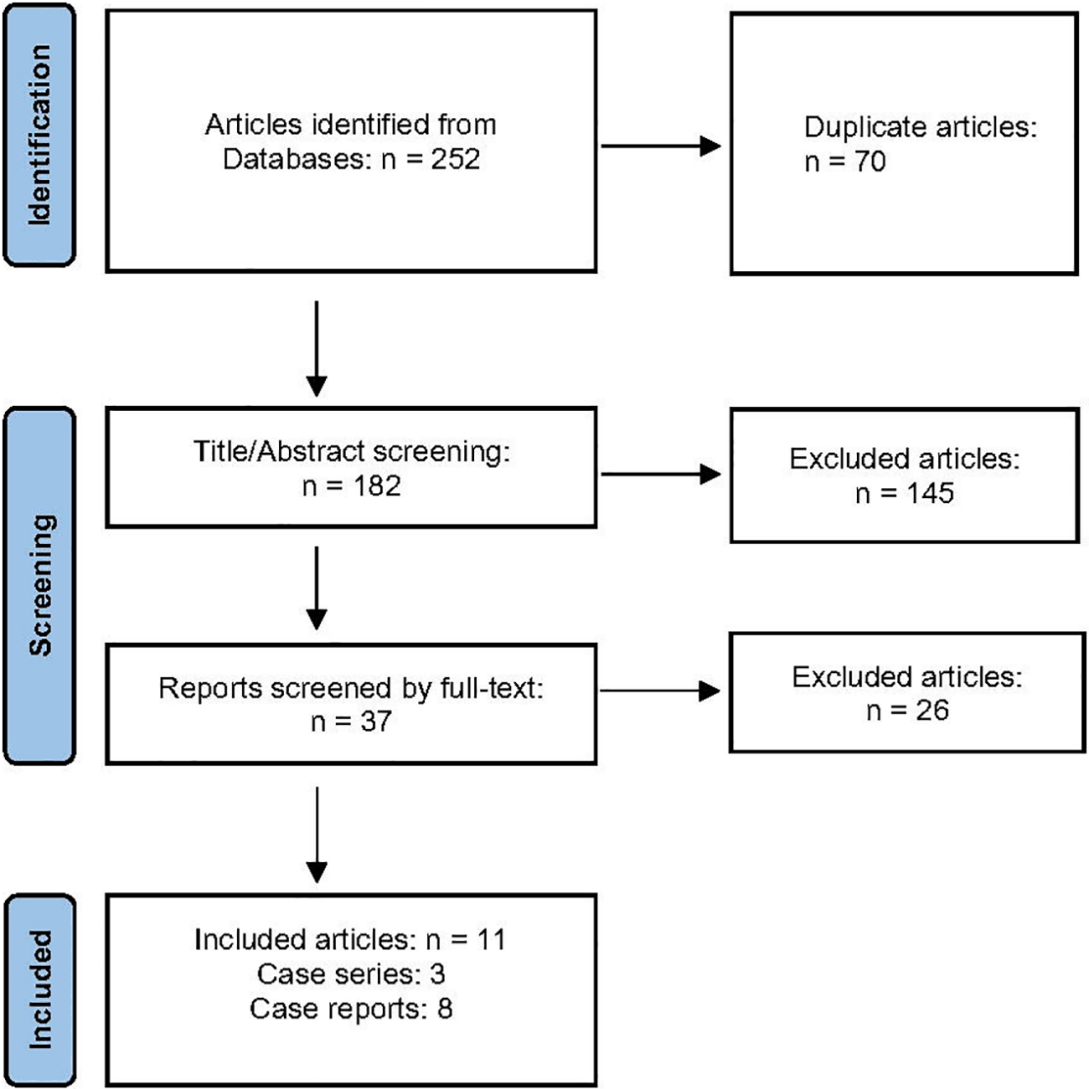
PRISMA flow chart of the eligible study selection.

We identified 252 articles in the initial search. After excluding duplicates (n = 70) and ineligible articles (Fig. 1), 11 articles (8 case reports^[8–15]^ and 3 case series^[6,16,17]^) containing 20 cases of COVID-19 vaccine-associated MG were included in our study. Regarding the articles’ quality, all 3-case series had a quality score >7 (Table S1, Supplemental Digital Content, http://links.lww.com/MD/J598). We reported these findings by case in Tables 1 and 2. These cases were reported from the UK (n = 7), Italy (n = 4), the USA (n = 2), South Korea (n = 2), Israel (n = 2), Croatia (n = 1), and Japan (n = 1).

**Table 1:** Study and patient characteristics by case.

#	Author	Country	Year	Sex	Age	Vaccine type	Vaccine dose	Time to onset (days)	MG type
1	Abicic et al	Croatia	2022	M	65	PfizerBioNTech	Booster	21	Oculobulbar
2	Chavez et al	USA	2021	M	82	PfizerBioNTech	2nd dose	2	Generalized
3	Fanella et al	Italy	2022	M	90	PfizerBioNTech	2nd dose	10	Oculobulbar
4				M	80	AstraZeneca	2nd dose	6	Oculobulbar
5				M	55	Spikevax	1st dose*	3	Generalized
6	Hoshina et al	Japan	2022	M	30	Spikevax	1st dose	2	Oculobulbar
7	Kang et al	S. Korea	2022	M	35	AstraZeneca	1st dose	7	Oculobulbar
8	Lee et al	S. Korea	2021	F	33	PfizerBioNTech	2nd dose	1	Generalized
9	Slavin et al	USA	2022	M	60	Spikevax	Booster	6	Generalized
10	Ramdas et al	UK	2022	F	13	PfizerBioNTech	1st dose	14	Generalized
11				M	59	AstraZeneca	1st dose	2	Generalized
12				M	63	PfizerBioNTech	Booster	3	Oculobulbar
13				M	73	PfizerBioNTech	Booster	12	Generalized
14				M	50	PfizerBioNTech	1st dose	7	Oculobulbar
15				F	83	PfizerBioNTech	1st dose	6	Generalized
16				M	77	AstraZeneca	1st dose	3	Generalized
17	Maher et al	Australia	2022	M	52	AstraZeneca	1st dose	1	Oculobulbar
18	Galassi et al	Italy	2022	M	73	AstraZeneca	1st dose	8	Oculobulbar
19	Watad et al	Israel	2021	M	72	PfizerBioNTech	2nd dose	1	Generalized
20				M	73	PfizerBioNTech	2nd dose	7	Generalized

MG = Myasthenia Gravis, S. Korea = South Korea, UK = United Kingdom, USA = United States of America.

* Symptoms worsened after the second dose.

**Table 2:** Findings, treatment, and clinical outcome by case.

#	Presenting symptoms	EMG	Seropositivity	Other findings	Treatment	Follow up
1	Binocular diplopia	NR	AChR + MuSK -	IM neostigmine test -	Pyridostigmine Corticosteroids	Improved after 1 month
2	Intermittent dysarthria	RNS +	AChR +	NR	Pyridostigmine Speech therapy	Initial improvement after 2 weeks Myasthenic crisis after 2 months Recovered and is rehabilitating
3	Asthenia, bilateral ptosis	RNS +	AChR +	NR	Pyridostigmine	Symptoms unchanged after 1 month
4	Binocular diplopia, bilateral ptosis, dysphagia	RNS +	AChR +	NR	Pyridostigmine Corticosteroids Azathioprine Plasma exchange	Improved after 2nd plasma exchange Mild ptosis at 3 months f/u
5	Upper limbs fatigability, binocular diplopia	RNS +	AChR +	NR	Pyridostigmine IVIg	At discharge: ptosis after 50s of upward gaze At 3-month f/u: upper limb fatigability after 90s of abduction
6	Binocular diplopia	NR	AChR + MuSK -	Iced-pack test + IM neostigmine test +	Pyridostigmine Corticosteroids	Symptoms improved but continue to fluctuate
7	Binocular diplopia	NR	AChR +	NR	NR	NR
8	Bilateral ptosis, Binocular diplopia	RNS +	AChR − MuSK −	IM neostigmine test +	Pyridostigmine	Partially improved after 4 days
9	Dysarthria, Binocular diplopia, dysphagia	RNS +	AChR −	NR	Pyridostigmine	Symptoms improved
10	NR	RNS +	AChR −	NR	Pyridostigmine Corticosteroids	NR
11	NR	NR	AChR +	NR	Pyridostigmine Corticosteroids	NR
12	NR	NR	AChR +	NR	Pyridostigmine	NR
13	NR	SFEMG +	AChR +	NR	Pyridostigmine Corticosteroids IVIg	NR
14	NR	RNS −	AChR +	NR	Pyridostigmine	NR
15	NR	RNS −	AChR +	NR	Pyridostigmine Corticosteroids IVIg	NR
16	NR	RNS + SFEMG +	AChR +	NR	Pyridostigmine Corticosteroids Plasma exchange	NR
17	Binocular diplopia	SFEMG +	AChR − MuSK −	Iced-pack test +	Pyridostigmine Corticosteroids	Partially improved
18	Left-sided ptosis	RNS +	AChR +	NR	Pyridostigmine	Symptoms improved
19	NR	RNS +	NR	NR	Corticosteroids Plasma exchange	Rapid response to treatment with quick symptom improvement
20	NR	RNS + SFEMG +	NR	NR	Pyridostigmine Plasma exchange	Intubated due to respiratory failure

AChR = acetylcholine receptor antibody, EMG = electromyography, IM = intramuscular, IVIg = intravenous immunoglobulin, MuSK = muscle specific kinase antibody, NL = normal, NR = not reported, RNS = repetitive nerve stimulation, SFEMG = single fiber electromyography.

The median (interquartile range) age was 64 (51, 75) years (ranging from 13 to 90 years), 9 patients were older than 70 years, and 17 out of the 20 patients were male (85%). In 14 patients, MG symptoms developed after receiving messenger RNA (mRNA)-based vaccines (Pfizer-BioNTech or Spikevax), and in the remaining 6 patients, after a vector-based vaccine (AstraZeneca). In patients with vector vaccine-associated MG, symptoms appeared mainly after the first dose (first/second/third doses (n): 5/1/0). However, a similar pattern was not observed in patients with mRNA vaccine-related MG (first/second/third doses (n): 5/5/4) (Table 1).

Data on the presenting symptom was reported for 11 patients; the most common symptoms, in order of frequency, were binocular diplopia (8/11), ptosis (4/11), dysarthria (2/11), dysphagia (2/11), and/or extremity asthenia (1/11). 5 out of 11 had more than one symptom. The median (interquartile range) time interval from vaccine injection to symptoms was 6 (2, 7.5), varying from 1 to 21 days. Regarding MG types, 9 patients had an oulobulbar MG, and the rest were diagnosed with generalized MG. All the female patients had oculobulbar MG (Table 1).

In 10 cases, imaging evaluations were performed to rule out other causes, including a brain computed tomography (CT) scan (n = 5), brain MRI (n = 5), and/or the chest CT scan (n = 6). The results of all imaging studies were normal, except for one case (patient #8) who exhibited mild thymic hyperplasia in chest CT scan.

An intramuscular neostigmine test was done for 3 patients (#1, #6, and #8) and was positive in 2 (patients #6 and #8). Two patients (#6 and #17) underwent an iced-pack test, and both experienced improvement in their symptoms, supporting the MG diagnosis (Table 2). Also, electromyography was performed in 15 patients, “only” repetitive nerve stimulation (RNS) in 11 (positive in 9 patients), “only” single fiber electromyography in 2 (positive in both), and RNS followed by single fiber electromyography in 2 (both: RNS + and SFEMG+, Table 2). The nerve used for RNS was reported in only five cases, facial nerve stimulation in 4 cases and orbicularis oculi in 1.

Serum levels of acetylcholine receptor antibody (AChR Ab) and/or muscle specific kinase antibody (MuSK Ab) antibodies were assessed in 18 patients; 14 only received an AChR Ab test (AChR Ab +: in 12), and 4 of both AChR and MuSK (AChR Ab + and MuSK Ab −: 2, AChR Ab − and MuSK Ab −: 2).

Regarding administrated medications (reported in 19 patients), 7 patients were treated with pyridostigmine alone, 5 with both pyridostigmine and corticosteroids, and 3 with intravenous immunoglobulin in combination with pyridostigmine and/or corticosteroids. Four patients underwent plasma exchange due to the severity of their condition and/or a lack of response to other treatments.

The clinical outcome was reported in 12 patients; 9 patients experienced complete improvement at discharge or 1-month follow-up, 2 experienced myasthenic crisis, and symptoms were unchanged in one of the patients 3 months after discharge.

## 4. Discussion

In our pooled cohort, we report 20 patients with Myasthenia Gravis following the administration of COVID-19 vaccines. Patients were predominantly male, nearly half of them were older than 70. In almost all patients, symptoms occurred less than 2 weeks after receiving the COVID-19 mRNA/vector-based vaccine. MG symptoms appeared after the vaccine’s first, second, or booster dose. On the contrary, most vector-based vaccine patients revealed symptoms after the first dose. Binocular diplopia and ptosis were the most common presenting symptoms. Almost all patients improved partially or completely in less than a month, but a myasthenic crisis developed in 2 patients.

New onset and a flare-up of MG have been reported following other vaccines, including influenza, hepatitis B, e-bacillus calmette-guérin, and human papillomavirus.^[17–22]^ The CDC/FDA vaccine adverse event reporting system recorded a total of 42 definite incident cases of postvaccination MG, most induced by influenza and hepatitis B vaccines, in adults in the United States from 1990 to 2017 (incidence rate of MG after vaccination: 2.1 per year).^[23]^

Overall, most MG patients are diagnosed between 10 and 70 years old,^[24,25]^ and usually, female patients (mean age: 28 years) are younger than male patients at the time of diagnosis (mean age: 42 years).^[26]^ Although no predominancy is reported regarding pure ocular MG, most generalized MG patients are female (female/male ratio: 3:2 or higher).^[27]^ More than 60% of MG cases following other vaccines were younger than 60 years old (mean age [SD]: 49.0 [18.9] years), and no gender predominancy was reported.^[23]^ In contrast, most MG cases associated with COVID-19 vaccines were male, and half were older than 70 years old (mean age [SD]: 60.9 [20.4] years).

Based on the Centers for Disease Control and Prevention/Food and drug association report on other vaccines, MG symptoms appeared over 6 weeks after vaccination (mean [SD]: 10 [10.7] days) in about 1 out of 5 cases.^[23]^ All reported COVID-19-associated MG, but one, occurred within 2 weeks after vaccine administration (mean [SD]): 6.1 (5.1) days; the exception was case #1, in whom the interval time was 21 days.

Regarding MG patients induced by other vaccines, one-third of newly diagnosed MG cases in the United States presented with a myasthenic crisis.^[23]^ The myasthenic crisis presented itself in none of the twenty COVID-19 vaccine-associated MGs. Still, 2 patients experienced a myasthenic crisis requiring intubation during hospitalization (patient #20) or 2 months after discharge (patient #2). Almost all other cases improved partially or completely during hospitalization or within 1 month after discharge.

The exact mechanism by which COVID-19 vaccines could induce autoimmune neuropathies remains unknown. However, the immunization processes following the vaccines could provoke an autoimmune response through establishing a special inflammatory environment.^[28]^ Molecular mimicry is one of the main proposed mechanisms; it is believed that the immune response would cross-react with self-antigens if vaccine antigens mimic self-antigens, leading to an autoimmune reaction. Another proposed mechanism is the Bystander effect, which hypothesizes that inflammatory signals can activate self-reactive T cells involved in the autoimmune processes during immune responses to vaccine antigens. Furthermore, the Epitope spreading mechanism could play a role in the autoimmune response to vaccines. Based on this mechanism, self-tissue damage during an inflammatory cascade could activate self-reactive T cells through the release of self-antigens. Additionally, the presence of double-stranded RNA or its analogs may play a key role in vaccine-associated MG through overexpressing IFN-b, a key agent of thymic events.^[3]^

Over half of the patients developed MG symptoms within 2 weeks after the first dose of the vaccine; some authors argued that vaccination likely exacerbated an already existing but asymptomatic form of MG in these patients rather than triggering a new onset of the disease. We believe that in cases with mild MG, it may be appropriate to consider administering the next dose of the vaccine. However, caution should be exercised. Given the potential risk associated with further exacerbation of MG, it may be advisable to change the type of vaccines in these cases. This approach could help minimize the potential impact on MG patients while still ensuring the benefits of vaccination against COVID-19.

## 5. Limitations and strengths

Regarding our study limitations, a few studies were available, and they mainly reported one case. Furthermore, our pooled data sets were incomplete for some variables, including laboratory tests, imaging, and electromyography. However, we included all relevant studies using a comprehensive search strategy to provide a best-evidence synthesis. To our knowledge, this is the first systematic review of reported cases of COVID-19 vaccine-associated MG.

## 6. Conclusion

High-risk individuals for developing MG following COVID-19 vaccination are likely males and adults older than 50. MG symptoms probably appear within 2 weeks after receiving the vaccine. MG associated with a vector-based COVID-19 vaccine is expected to occur after the first dose. The presenting symptoms in COVID-19 vaccine-related MG are possibly similar to MG induced by other causes. Most patients are expected to experience partial or complete improvement within a month, but the occurrence of a myasthenic crisis is probable. More extensive studies are necessary to draw definitive conclusions regarding the association or causality between COVID-19 vaccines and MG, and to elucidate the specific mechanisms by which these vaccines may induce such autoimmune conditions.

## Acknowledgments

Researchers appreciated the Clinical Research Development Units of Kamali and Rajaei Hospitals at Alborz University of Medical Sciences.

## Author contributions

**Conceptualization:** Amir Hossein Tayebi, Hadith Rastad.

**Data curation:** Parham Samimisedeh, Aryan Ayati.

**Formal analysis:** Elmira Jafari Afshar, Aryan Ayati, Laleh Foroutani.

**Investigation:** Aryan Ayati.

**Methodology:** Parham Samimisedeh, Elmira Jafari Afshar.

**Project administration:** Amir Hossein Tayebi.

**Resources:** Elaheh Ghalehnovi.

**Software:** Parham Samimisedeh, Elmira Jafari Afshar.

**Supervision:** Nahid Abbasi Khoshsirat, Hadith Rastad.

**Validation:** Elmira Jafari Afshar, Nahid Abbasi Khoshsirat, Hadith Rastad.

**Visualization:** Elaheh Ghalehnovi.

**Writing – original draft:** Amir Hossein Tayebi, Parham Samimisedeh, Aryan Ayati, Elaheh Ghalehnovi.

**Writing – review & editing:** Laleh Foroutani, Nahid Abbasi Khoshsirat, Hadith Rastad.

## Supplementary Material
