# Supplementary material for: Clinical features and outcomes of Myasthenia Gravis associated with COVID-19 vaccines: A systematic review and pooled analysis
Source: Medicine (Baltimore). 2023 Oct 6;102(40):e34890. doi: 10.1097/MD.0000000000034890 (PMC10553155; doi:10.1097/MD.0000000000034890)
Supplement: Supplementary file 1 [file medi-102-e34890-s001.docx]

| **Supplementary table 1.** Quality assessment of the included case series based on the JBI checklist for case series | | | |
| --- | --- | --- | --- |
| **Items** | **Watad et al. (2021)** | **Fanella et al. (2022)** | **Ramdas et al. (2022)** |
| Were there clear criteria for inclusion in the case series? | + | + | + |
| Was the condition measured in a standard, reliable way for all participants included in the case series? | + | + | + |
| Were valid methods used for identification of the condition for all participants included in the case series? | + | + | + |
| Did the case series have consecutive inclusion of participants? | + | + | + |
| Did the case series have complete inclusion of participants? | - | + | + |
| Was there clear reporting of the demographics of the participants in the study? | + | + | + |
| Was there clear reporting of clinical information of the participants? | - | + | - |
| Were the outcomes or follow-up results of cases clearly reported? | - | + | - |
| Was there clear reporting of the presenting site(s)/clinic(s) demographic information? | + | + | - |
| Was statistical analysis appropriate? | + | + | + |
| Total score | 7 | 10 | 7 |
